# Identifying the impact of social isolation and loneliness on psychological well-being among the elderly in old-age homes of India: the mediating role of gender, marital status, and education

**DOI:** 10.1186/s12877-023-04384-1

**Published:** 2023-10-20

**Authors:** Bijeta Mishra, Jalandhar Pradhan, Suman Dhaka

**Affiliations:** 1 National Institute of Technology, Rourkela, Odisha India; 2 Indian Institute of Technology, Jodhpur, Rajasthan India

**Keywords:** Social isolation, Loneliness, Psychological well-being, Older adults

## Abstract

**Background:**

Social isolation and loneliness can be detrimental to the overall functioning of the older adults. The study examines the impact of social isolation and loneliness on the psychological well-being of older adults residing in various old-age homes in India and investigates the mediating role of gender, marital status, and education level in the way social isolation and loneliness affect psychological well-being.

**Methods:**

Data has been collected from 320 individuals aged sixty years or above. Data were collected using standardized measures like Lubben Social Network Scale- 6, revised UCLA Loneliness Scale, and shortened version of psychological well-being scale by Ryff & Keyes (1995). Multivariate and mediation analysis were performed to understand the associations of social isolation and loneliness with psychological well-being.

**Results:**

A statistically significant MANOVA effect was obtained for social isolation (F = 3.836, p < .01), and loneliness (F = 3.782, p < .01). Gender and education as independent factors were significantly associated with the psychological well-being of individuals. However, both gender and education did not mediate the impact of social isolation and loneliness on the psychological well-being of older adults. Further, marital status had a partial mediating effect on the relationship between social isolation, loneliness, and psychological well-being.

**Conclusions:**

The findings of the study can be incorporated into measures aiming at alleviation of feelings of social isolation and loneliness among the elderly. Further, the findings can be used to design various intervention strategies aimed at the reduction of social isolation and loneliness among older adults and the restoration of their psychological well-being.

## Introduction

Senescence or old age is often accompanied by an array of concerns like declining health, cognitive abilities and social connectedness, reduced mobility, loneliness, and increased social isolation challenging their overall health and well-being [1–4]. The World Health Organization has emphasized on the importance of healthy aging [5], with a comprehensive approach of well-being that incorporates physical, psychological, and social dimensions. Social isolation and loneliness have been identified as important determinants of well-being and overall functioning among the aged [6–8]. With the increasing geriatric population and changing socio-cultural context, there has been an increase in social isolation and loneliness among the older adults [9]. Social isolation is an objective phenomenon which is often defined as “a state where individuals have minimal contacts and paucity of social relationships and social engagements” [10]. Furthermore, loneliness is often defined as “a discrepancy between an individual’s desired and achieved levels of social relations” [11]. It is often understood as a discrepancy of an individual’s actual and desired state of social relationships and relationship quality [12].

India is home to one-fourth of the global geriatric population; the elderly population will rise to 319 million by 2050 [13]. According to the Ministry of Statistics & Programme Implementation, Government of India, 2021, approximately 13% of the population in India will comprise adults aged 60 years or above [14]. The prevalence of social isolation among elderly in India is almost 34%, and research evidence cites a rising trend of this phenomenon [15]. Further, prevalence of loneliness among Indian older adults is also alarming with almost 55.4% of older adults experiencing loneliness [16]. Social isolation and loneliness are serious yet underrated phenomena among older adults across the globe that pose health risk threats. Socially isolated and lonely individuals can be affected physically and psychologically, thereby affecting their optimal functioning [17].

### Psychological well-being, social isolation, and loneliness

Successful aging incorporates the idea of growing old with a positive orientation towards life consisting of good health, functional capacity, autonomy, acceptance of self and others, deriving a sense of purpose in life. Engaging oneself in healthy social interactions is instrumental in achieving these goals, thereby fostering psychological well-being in the declining years of life. Psychological well-being is a positive psychological construct related to subjective views of oneself and life [18]. The multidimensional model of psychological well-being (PWB) encompasses six components: the meaning, purpose, and direction people give to their lives; autonomy; personal growth; mastery over one’s environment; maintaining positive relationships; self-knowledge and self-acceptance. Furthermore, Seligman (2012), in his PERMA (Positive Emotion, Engagement, Relationships, Meaning, and Accomplishment) model of psychological well-being, has focused on engagement and social relationships as two key attributes to achieving psychological well-being [19]. According to this theory, engaging in meaningful and pleasure-eliciting activities enhances the number of positive neurotransmitters and hormones, thereby improving an individual’s sense of well-being. The model suggests that healthy relationships are basic human needs that are crucial for a meaningful life. Therefore, maintaining positive and meaningful social relationships foster well-being among individuals. These relationships promote love, intimacy, and strong emotional and physical interaction with other human beings and thereby enhance the resilience capacity of the individuals which leads to improved psychological well-being. However, social isolation and loneliness pose a threat to their psychological well-being by hampering the number and quality of relationships and creating disequilibrium in the feelings of well-being. Research findings have consistently advocated the association of perceived social connectedness with enhanced health and psychological well-being in individuals [20, 21].

### Psychological well-being of older adults in old-age homes

Research evidences have supported the importance of psychological well-being in reduced incidence of diseases and premature mortality [22]. Additionally, psychological well-being has also been associated with better cognitive functioning of the older adults [23]. However, older adults residing in old-age homes have limited choice in terms of relocation autonomy which in turn results in lowered levels of psychological well-being [24]. Moreover, increased levels of loneliness in case of older adults residing in old-age homes can also be attributed as one of the major causes for reduced psychological well-being [25]. Furthermore, factors like care of family members, appropriate medical facilities, financial stability have been related to higher psychological well-being. Inadequacy of these factors for the residents of old-age homes affects their psychological well-being negatively [26].

With the changing social dynamics and patterns of social interactions, there has been an increased level of social isolation and loneliness among elderly individuals. In the Indian context, which is perceived as collectivistic in nature, the joint family system has prevailed over the years, where the older adults have had a pivotal role to play. Consequently, social isolation and loneliness experienced was less prevalent amongst the older adults. However, with the recent shifts in the societal paradigms from joint family systems to nuclear family systems, the societal roles assigned to the older adults have also changed, making them lonely and socially isolated. Further, the number of older adults residing in old-age homes have increased significantly over the recent years, leading to increased levels of social isolation and loneliness in the aged population in the Indian context. Old-age homes can be defined as residences for the older adults that provide facilities like assisted living and nursing and cater to the needs and concerns of the aged [27]. The Department of Social Justice and Empowerment, Government of India aims to setup an old age home in every district of all states across the country under Integrated Programme for Senior Citizens scheme **(**IPSrC). According to Ministry of Social Justice & Empowerment (2022), there are 502 old-age homes under IPSrC in different states across the country. Moreover, Govt. of India provides grant-in-aid under Atal Vayo Abhuday Yojana (AVYAY) to various Non-Government Organisations (NGOs), and voluntary organisations for managing old-age homes. They are directed to follow set of norms in terms of the living conditions to ensure a healthy life for the aged population. There are directives regarding living space comprising of minimum area for bedroom per resident (7.5 square meters), total carpet area per resident is suggested to be 12 square meters, hygienic toilet and bathing facilities (one for every ten residents), and separate beds for each resident. Along with the above-mentioned mandates various other facilities like adequate water for drinking and other purposes, adequate nutrition and medical facilities (first aid, monitors for diabetes and blood pressure, regular health check-up, medication), electricity and fans, dining halls, kitchen-cum-store rooms, clothing (four pairs per year), and recreation facilities like television, newspapers, and books [28].

Though research evidence has studied the impact of social isolation and loneliness as individual constructs, the association among these factors is less explored. Moreover, there is a dearth of research to understand the impact that social isolation and loneliness can have on the psychological well-being of the geriatric group. In addition to this, the experiences of social isolation and loneliness might vary based on gender, given the difference in assigned societal roles [29, 30]. Further, if factors like educational status of the older adults, and their marital status have a role to play in the way social isolation and loneliness impact psychological well-being of the older adults, it is less explored. These aspects necessitate the need to assess the impact of loneliness and social isolation on psychological well-being of the elderly as it detrimentally affects their overall functioning and quality of life.

### Objectives

The current study aims to assess the underlying relationship among social isolation and loneliness and tried to assess the co-existence of the two factors. Further, the paper evaluates the impact of social isolation and loneliness on the psychological well-being of the older adults residing in various old-age homes in India. Moreover, the study aims to explore the impact of social isolation and loneliness on the individual constructs of psychological well-being among the older adults. The study purports to find out if factors like gender, marital status, and educational status have a mediating role to play in the way social isolation and loneliness affect the psychological well-being of the older adults.

### Methodology

#### Participants

Older adults residing in various old-age homes were selected for the study through the process of purposive sampling. The older adults residing in old-age homes differ from their counterparts staying at their homes on various aspects. Research evidence report older adults staying at old-age homes are dissatisfied with the food provided, and express inability to pursue hobbies. Additionally, older adults staying with family have higher levels of autonomy, intimate relationships, and social participation as compared to the residents of old-age homes [31]. The participants had relocated to old-age homes citing reasons of either or all the factors like physical and psychological ailments, lack of social and emotional support from family members and friends, and financial insecurities [32]. Initially, 385 participants were shortlisted for the study. The records of residing individuals were checked and the older adults who were diagnosed with clinical problems like depression, anxiety, schizophrenia, dementia were excluded from being a part of the study. Individuals with intellectual disabilities were also excluded from the study. Further, terminally ill older adults suffering from diseases like Cancer, Coronary Heart Diseases, Parkinson’s, etc. were also not taken into consideration for study purposes. Therefore, a total of 320 participants aged sixty years or above were interviewed for the study.

The participants were categorised based on various socio-demographic characteristics like gender, level of education, and marital status. The sample consisted of both males (n = 151) and females (n = 169). The participants were further categorised based on their level of education. Participants who did not complete primary level of education were categorised as ‘uneducated,’ whereas participants who had completed primary education and above were categorised as ‘educated.’ They were also categorised based on their marital status namely ‘currently married,’ ‘widowed,’ ‘divorced/separated/deserted’, and ‘never married’. Each participant was briefly explained about the study, and they were interviewed after receiving an informed consent from them.

#### Measures

##### Social isolation

Lubben Social Network Scale-6 (LSNS- 6), was used to assess social isolation among older adults [33]. The items assess the social interactions an individual has with family members and friends, including how often the participant hears from his/her family and friends, and how many friends or family members he/she feels is close enough to share. It consists of six items which are rated on a six-point Likert Scale ranging from 1 (None) to 6 (Nine or more). A score > 12 is interpreted as ‘at-risk’ social isolation.

##### Loneliness

The Revised UCLA Loneliness Scale was used to assess the subjective feelings of loneliness [34]. The scale consists of twenty items, out of which eleven items are positively worded and nine items are negatively worded. It is measured on a four-point Likert scale with scores as 1(Never), 2(Rarely), 3(Sometimes) to 4 (Often). The range of scores varies from 20 to 80. Individuals scoring above 40 are considered as lonely.

##### Psychological well-being

The shortened version of the Psychological Well-Being Scale comprising 42 items by Ryff & Keyes (1995) was used to assess psychological well-being [35]. The test measures psychological well-being on six dimensions namely, autonomy, environmental mastery, personal growth, positive relations with others, purpose in life, and self-acceptance. The items are rated on a six-point Likert Scale, where a score of ‘1’ represents ‘strongly disagree’ and a score of ‘6’ represents ‘strongly agree’. Higher scores are indicative of higher levels of well-being.

## Results

Descriptive statistics were reported as means and standard deviations of the groups. Multivariate analysis was carried out to analyse the effect of social isolation and loneliness on the overall psychological well-being as well as the individual constructs of psychological well-being. Further, mediation analysis was carried out to examine the role of important socio-demographic characteristics like gender, education, and marital status in the impact of social isolation and loneliness on the psychological well-being of the older adults.

The participants have been categorised based on socio-demographic dimensions like gender, education status, marital status (Table 1). Males comprise 47.19% of the total sample, whereas 52.81% of the participants are females. Further, almost 46.25% (n = 148) participants are educated, whereas 53.75% participants are uneducated. Based on marital status participants have been categorised as ‘currently married’ (31.88%), ‘widowed’ (52.19%), ‘divorced/separated/deserted’ (5.94%), and ‘never married’ (10.00%). Moreover, based on the scores of social isolation, and loneliness, 84.38%(n = 270) participants are socially isolated, and 86.88% (n = 278) are lonely (Table 2).

**Table 1:** Frequency Table for Participants’ Socio-Demographic Variables

Variables	n (%)	
**Gender**		
Males	151 (47.19)	
Females	169 (52.81)	
**Education**		
Educated	148(46.25)	
Uneducated	172(53.75)	
**Marital Status**		
Currently Married	102(31.88)	
Widowed	167(52.19)	
Divorced/ Separated/ Deserted	19 (5.94)	
Never Married	32(10.00)	

*Source: Authors’ Calculation*

**Table 2:** Descriptive Statistics of Categorical Variables

Variables	n (%)	Mean (SD)	
**Social Isolation**		1.15(0.363)	
High Social Isolation	270(84.38)		
Low Social Isolation	50(15.63)		
**Loneliness**		1.13(0.338)	
High Loneliness	278(86.88)		
Low Loneliness	42(13.13)		

*Source: Authors’ Calculation*

Table 3 presents the mean and standard deviations of the various groups of participants on the dimension of psychological well-being as well as the individual components of psychological well-being. Older adults who had high social isolation and high levels of loneliness had the least scores on overall psychological well-being (M = 109.21, SD = 24.97), as well as for all the components of psychological well-being. Contrarily, participants who reported low on social isolation and loneliness had the best overall psychological well-being (M = 144.57, SD = 28.14). They also exhibited better results in individual components of psychological well-being as compared to their socially isolated and lonely counterparts.

**Table 3:** Descriptive Statistics of Group of Participants on Dimension of Components of Psychological Well-being

Group			Mean(SD)						
Social Isolation	Loneliness	n	Psychological Well-Being	Autonomy	Environmental Mastery	Personal Growth	Positive Relations	Purpose in Life	Self-Acceptance
High Social Isolation	High Loneliness	242	109.21(24.97)	16.69(6.24)	17.93(3.82)	19.27(4.86)	16.87(6.87)	23.07(3.32)	15.38(5.46)
	Low Loneliness	28	129.18(22.30)	19.18(5.49)	20.43(3.69)	22.18(4.33)	25.00(6.60)	24.50(3.49)	17.89(4.88)
Low Social Isolation	High Loneliness	36	128.67(37.16)	18.94(6.49)	20.08(4.44)	22.08(6.90)	24.44(10.96)	24.86(4.07)	18.25(7.32)
	Low Loneliness	14	144.57(28.14)	23.93(7.39)	20.79(4.93)	23.93(5.59)	27.29(7.21)	26.64(4.36)	22.00(8.09)

*Source: Authors’ Calculation*

**Fig. 1:**
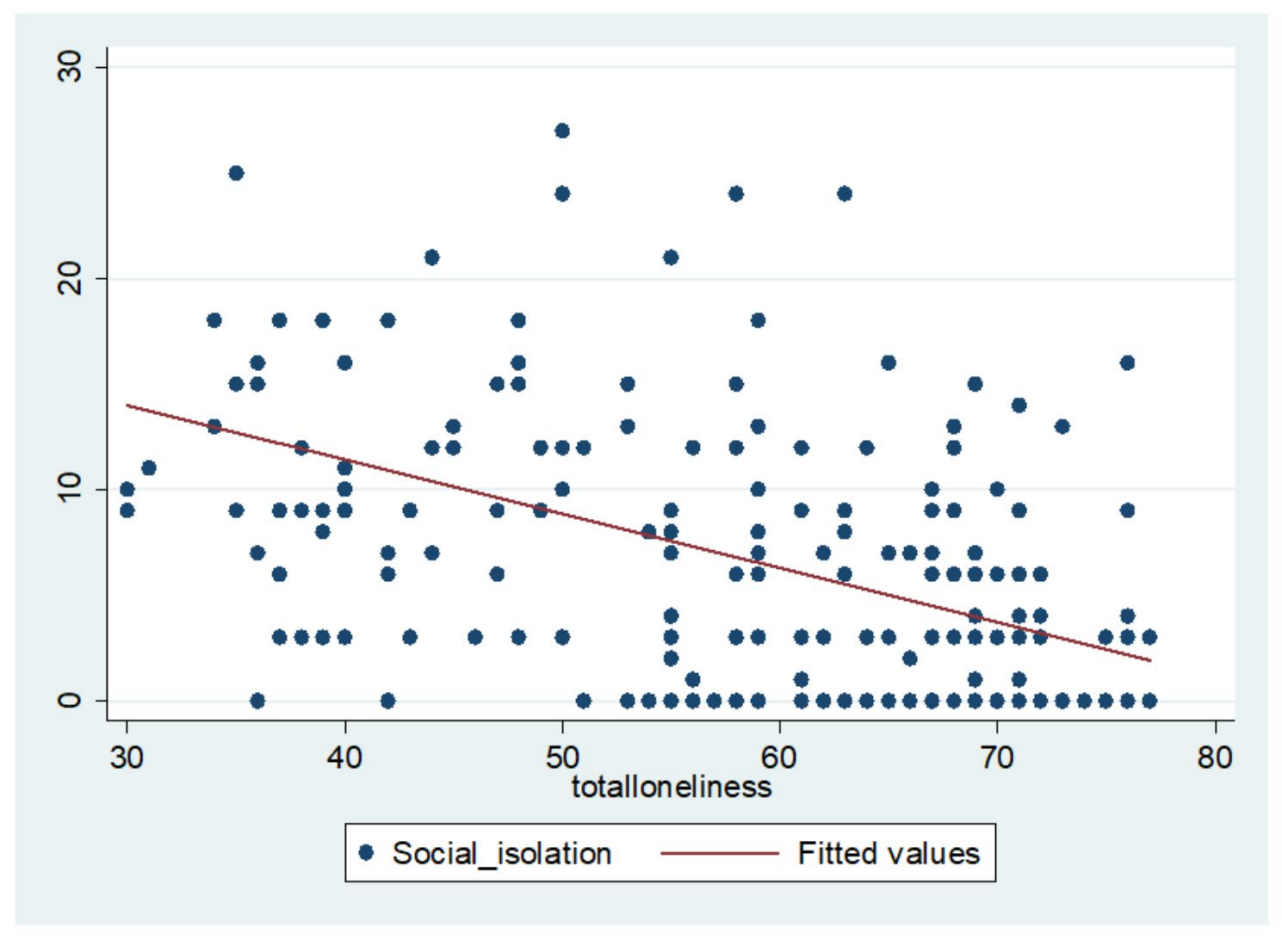
Relationship between Loneliness and Social Isolation Source: Authors’ Calculation

Furthermore, the independent variables, namely social isolation and loneliness exhibited a significant negative correlation (r=-.505**), (Fig. 1) suggesting that with lower scores of social isolation, which is indicative of heightened levels of social isolation, loneliness tends to increase. Hence to further understand the co-existence of social isolation and loneliness, linear regression analysis was performed (Table 4). A significant regression equation was obtained (F= (1,318) = 109.231, p < .01), with an R^2^ of 0.256. Therefore, social isolation was a significant predictor of loneliness and explained 25.6% of the variance.

**Table 4:** Association Between Social Isolation and Loneliness

Model	R^2^	*B*	β	t-value	F
Loneliness	0.256	− 0.995	− 0.506**	-10.451	109.23

Note: ** represent 1% level of significance

*Source: Authors’ Calculation*

Multivariate analysis (Table 5) was carried out to examine the impact of social isolation and loneliness on psychological well-being of the participants. It was conducted to assess if there are significant differences in psychological well-being among the participants experiencing varying levels of social isolation and loneliness. Wilk’s Lambda test statistic was used for the analysis. A statistically significant MANOVA effect was obtained for social isolation (F = 3.836, p < .01), with the multivariate effect size being approximately 0.07, suggesting that almost 7% of the variance was explained by social isolation while determining the attributing factors of psychological well-being among older adults. Furthermore, loneliness (F = 3.782, p < .01) also had a significant effect on psychological well-being and played a vital role in determining psychological well-being among the participants, with partial η^2^ being 0.068, suggesting almost 7% of the variance was explained by loneliness. As the interaction effect of the independent variables – that is, social isolation and loneliness – were not statistically significant; the main effects of social isolation, and loneliness were considered for the analysis.

**Table 5:** Summary of Multivariate Analysis of Variance

Effect	Value	F	Hypothesis df	Error df	Partial η^2^
Intercept	0.042	1191.569**	6	311	0.958
Social Isolation Loneliness Social Isolation*Loneliness	0.931 0.932 0.962	3.836** 3.782** 2.050	6 6 6	311 311 311	0.069 0.068 0.038

Note: ** represent 1% level of significance

*Source: Authors’ Calculation*

Furthermore, Test of Between-Subject Effects (Table 6) were analysed to determine how social isolation and loneliness affect the overall psychological well-being, as well as the individual components of psychological well-being of the participants. Results showed that social isolation (F = 12.412, p < .01, partial η^2^ = 0.038) had a significant impact on the overall psychological well-being, explaining almost 4% variance. Further, social isolation had a significant impact on various components of psychological well-being, namely autonomy, personal growth, positive relations, purpose in life, self-acceptance. However, it did not impact environmental mastery significantly, suggesting that social isolation does not act as deciding factor in determining older adults’ ability to manage and modify the environmental factors and activities in accordance with their needs and benefits. Moreover, loneliness had a significant impact on the overall psychological well-being (F = 13.152, p < .01, partial η^2^ = 0.040) as well as the deconstructed factors of psychological well-being. It was observed that older adults who are not socially isolated and experience lower levels of loneliness exhibit best psychological well-being (M = 144.57, SD = 28.14), encompassing the individual components.

**Table 6:** *Summary of Effect of Independent Variables on Dependent Variables*

Source	Dependent Variable	df	F	Partial η^2^
Social Isolation	Psychological Well-being	1	12.412**	0.038
	Autonomy	1	8.997**	0.028
	Environmental Mastery	1	2.927	0.009
	Personal Growth	1	5.707**	0.018
	Positive Relations	1	12.659**	0.039
	Purpose in Life	1	9.190**	0.028
	Self-Acceptance	1	10.482**	0.032
Loneliness	Psychological Well-being	1	13.152**	0.040
	Autonomy	1	10.240**	0.031
	Environmental Mastery	1	4.755**	0.015
	Personal Growth	1	6.194**	0.019
	Positive Relations	1	15.673**	0.047
	Purpose in Life	1	6.125**	0.019
	Self-Acceptance	1	8.447**	0.026
Social Isolation* Loneliness	Psychological Well-being	1	0.169	0.001
	Autonomy	1	1.142	0.004
	Environmental Mastery	1	1.500	0.005
	Personal Growth	1	0.309	0.001
	Positive Relations	1	3.641	0.011
	Purpose in Life	1	0.074	0.000
	Self-Acceptance	1	0.327	0.001

Note: ** represent 1% level of significance

*Source: Author’s Calculation*

**Fig. 2:**
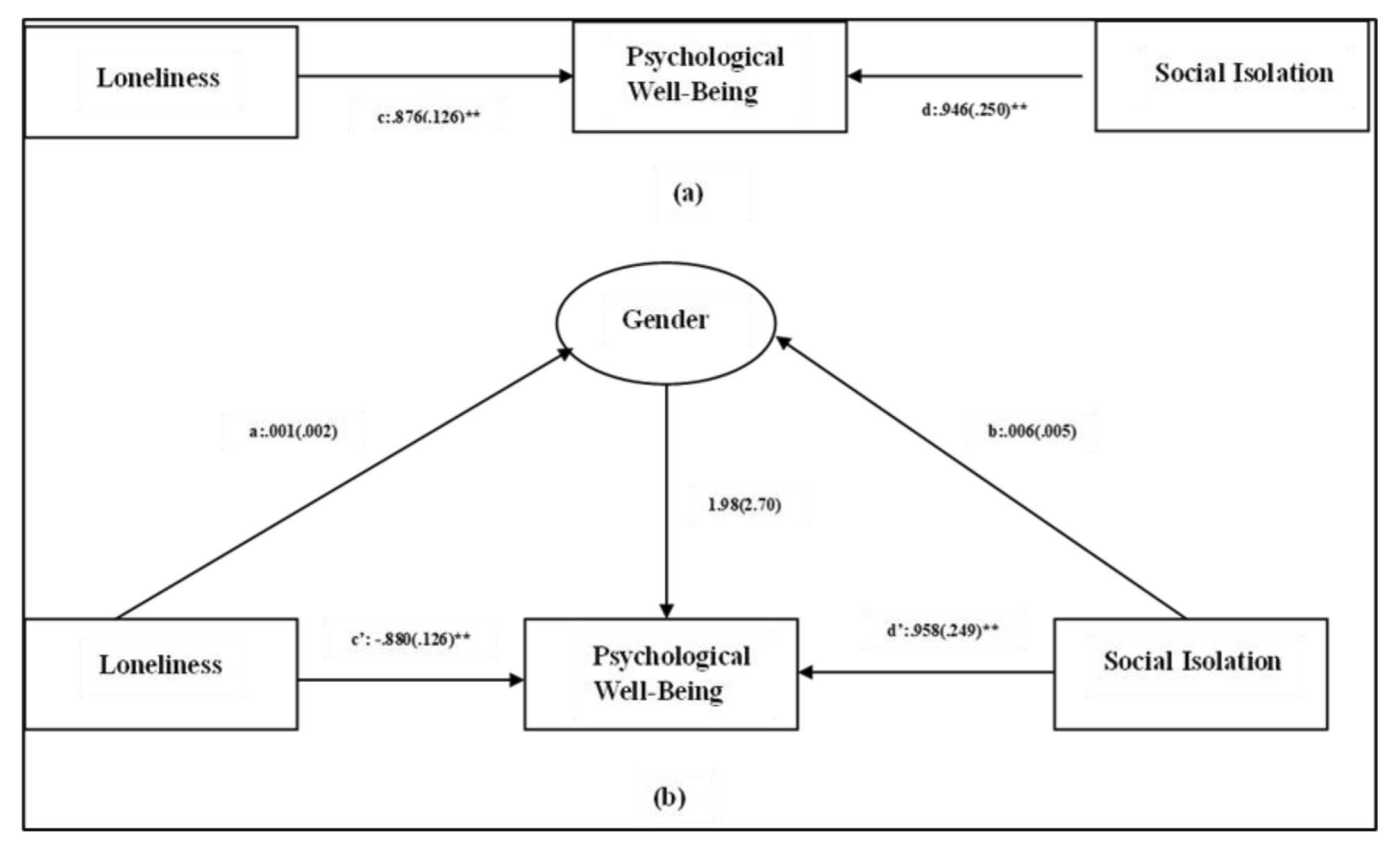
Mediating role of gender in the impact of social isolation and loneliness on psychological well-being. **Note:** **(a)** Direct relationship between loneliness, psychological well-being and social isolation. **(b)** Total relationship between loneliness, psychological well-being and social isolation, depicting the mediation effect of gender. Notes: Values outside parentheses = path coefficient or unstandardized coefficient; values in parentheses = standard error, **= p<.01. The model fit statistics were as follows: chi-square =106.19, df=1 Source: Authors’ Calculation

**Fig. 3:**
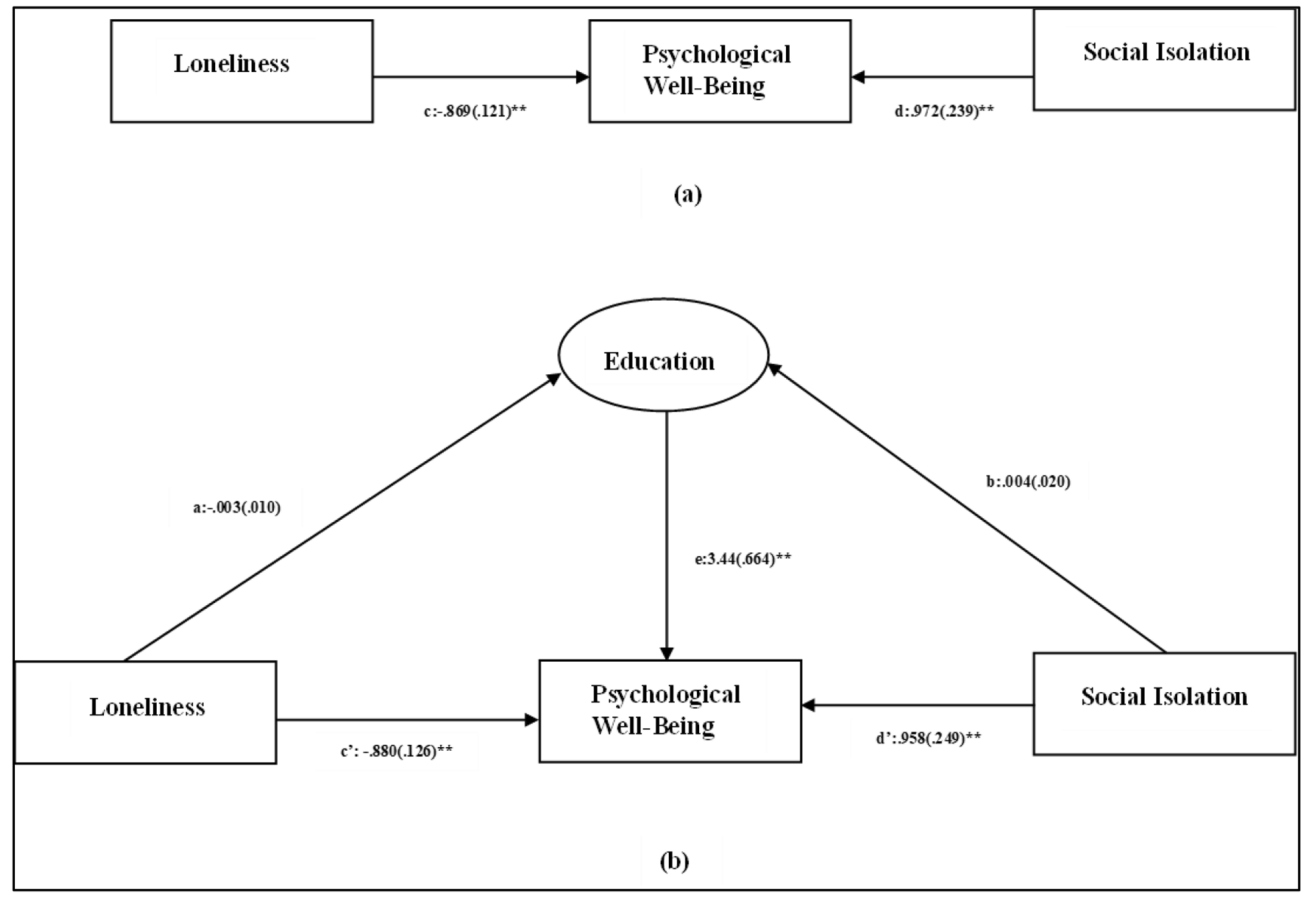
Mediating role of education in the impact of social isolation and loneliness on psychological well-being. **Note: (a)** Direct relationship between loneliness, psychological well-being and social isolation. **(b)** Total relationship between loneliness, psychological well-being and social isolation, depicting the mediation effect of education. Notes: Values outside parentheses = path coefficient or unstandardized coefficient; values in parentheses = standard error, **= p<.01. The model fit statistics were as follows: chi-square =128.21, df=1 Source: Authors’ Calculation

**Fig. 4:**
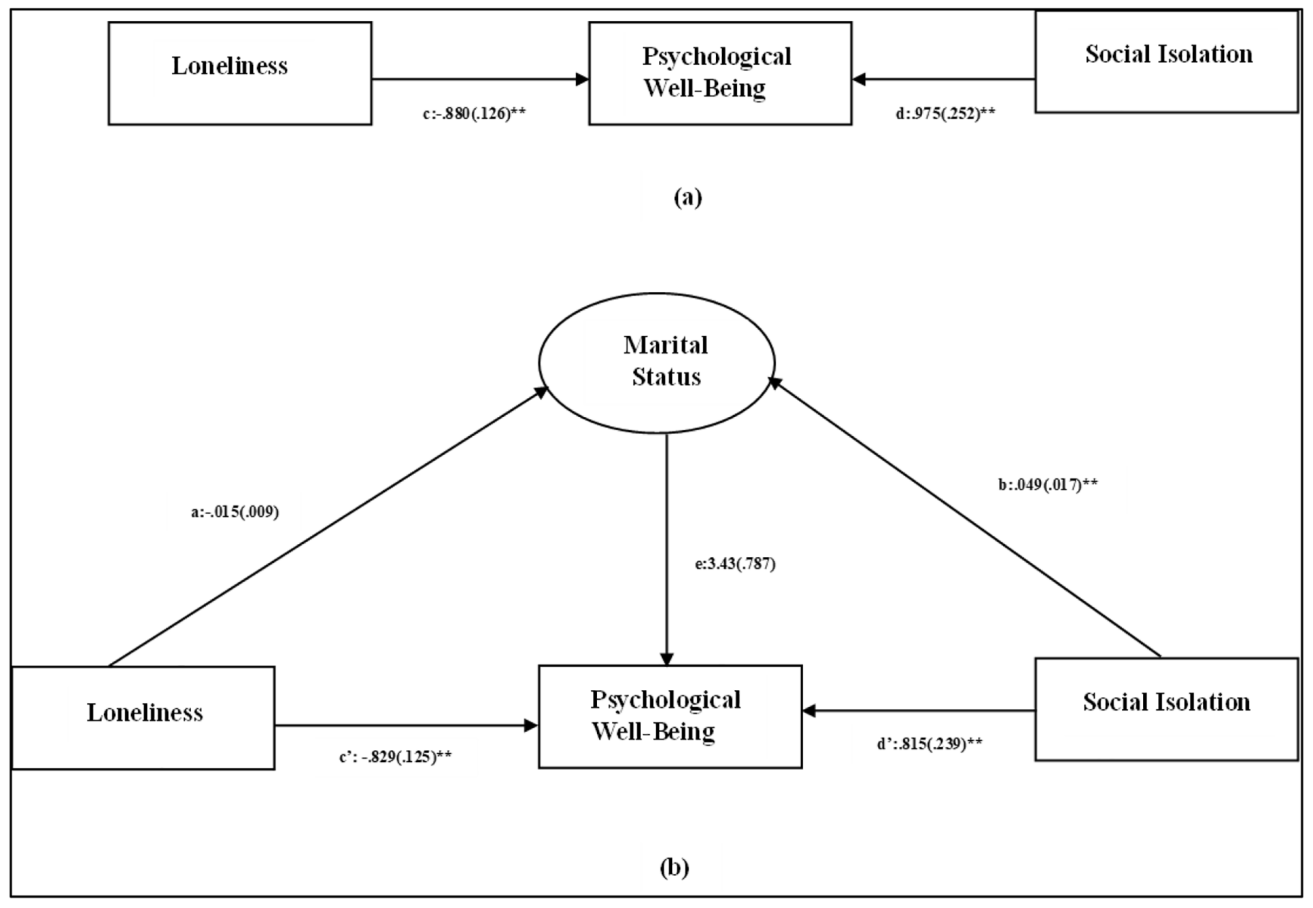
Mediating role of marital status in the impact of social isolation and loneliness on psychological well-being. **Note:(a)** Direct relationship between loneliness, psychological well-being and social isolation. **(b)** Total relationship between loneliness, psychological well-being and social isolation, depicting the mediation effect of marital status. Notes: Values outside parentheses = path coefficient or unstandardized coefficient; values in parentheses = standard error, *= p<.05, **= p<.01. The model fit statistics were as follows: chi-square =113.396, df=1 Source: Authors’ Calculation

Mediation analysis was performed to assess the mediating role of factors like gender, education, and marital status, in the relationship between social isolation, loneliness, and psychological well-being. The results have been presented in Table 7. Further, the path diagrams (Figs. 2 and 3, and 4) illustrate direct effects as well the total effects in the form of regression coefficients of the independent variables and mediators on the dependent variable, i.e., psychological well-being.

**Table 7:** Direct, indirect, and total effects of independent variables

			Gender					Education					Marital status				
						95% Confidence Interval					95% Confidence Interval					95% Confidence Interval	
			Estimate	Std. Error	z-value	Lower	Upper	Estimate	Std. Error	z-value	Lower	Upper	Estimate	Std. Error	z-value	Lower	Upper
**Direct Effects**																	
Loneliness	→	Psychological Well-being	− 0.876	0.126	-6.91**	-1.125	− 0.628	− 0.869	0.121	-7.13**	-1.108	− 0.630	− 0.880	0.126	-6.94	-1.12	− 0.63
Social Isolation	→	Psychological Well-being	0.946	0.250	3.78**	0.456	1.436	0.972	0.239	4.05**	0.502	0.442	0.975	0.252	3.86**	0.480	1.470
**Indirect Effects**																	
Loneliness	→	Psychological Well-being	− 0.003	0.006	− 0.480	− 0.016	0.010	− 0.010	0.035	-0.30	− 0.079	0.058	0.051	0.002	0.51	− 0.005	0.007
Social Isolation	→	Psychological Well-being	0.012	0.019	0.620	− 0.025	0.049	0.013	0.069	-0.20	− 0.15	0.122	− 0.16	0.029	-0.62*	− 0.083	0.051
**Total Effects**																	
Loneliness	→	Psychological Well-being	− 0.880	0.126	-6.93**	-1.12	− 0.631	− 0.880	0.126	-6.93**	-1.128	− 0.631	− 0.829	0.124	-6.43	− 0.1.10	− 0.65
Social Isolation	→	Psychological Well-being	0.958	0.249	3.84**	0.469	1.44	0.958	0.249	3.84**	0.469	1.44	0.815	0.239	3.72**	0.458	1.401

Note: ** represent 1% level of significance

*Source: Author’s Calculation*

The results (Table 7) revealed that direct effect of both social isolation, and loneliness on psychological well-being were statistically significant, suggesting that social isolation and loneliness can be considered as determining factors of psychological well-being. However, both the factors namely social isolation (β_1_ = 0.012, z_1_ = 0.62, p_1_ > 0.05) and loneliness (β_2_ = − 0.003, z_2_ = − 0.48, p_2_ > 0.05.) had a non-significant indirect impact while assessing the role of gender as a mediating factor. This implies that gender does not impact the effect that social isolation and loneliness have on the psychological well-being of the older adults. Furthermore, education had a significant impact on the psychological well-being of the older adults (e = 3.44, z = 5.19, p < .01). This is indicative of the fact that education as an independent factor has a role to play in determining the psychological well-being of the older adults. However, it does not mediate the impact of social isolation and loneliness on the psychological well-being. This is reflected in the non-significant indirect effect of social isolation (β_1_ = 0.013, z_1_ = − 0.20, p_1_ > 0.05) and loneliness (β_2_ = − 0.010, z_2_ = − 0.30, p_2_ > 0.05.) on the psychological well-being with education as a mediating factor. It was observed that marital status did not have significant impact on the psychological well-being of the older adults. This implies that marital status as an independent construct does not have a protective impact on the psychological well-being of the older adults. However, both social isolation (β_1_=-0.16, z_1_= -0.62, p_1_ < 0.05) and loneliness (β_2_ = 0.051, z_2_ = 0.51, p_2_ < 0.05) exhibit statistically significant indirect effect on psychological well-being with marital status as the mediating factor. This suggests that marital status partially mediates the association of social isolation and loneliness with the psychological well-being of the older adults. Here, β_1,_ z_1,_ and p_1_ denote the regression coefficient, z score, and probability values for social isolation, and β_2_, z_2_, p_2_ are indicative of the regression coefficient, z score, and probability values for loneliness. Further, the total effect of the afore-mentioned independent variables on psychological well-being was reported to be significant, suggesting the importance of assessing social isolation and loneliness as determinants of psychological well-being.

## Discussion

With the rising number of older adults residing in old-age homes, it has become imperative to investigate the aspects ensuring optimal functioning of the elderly. Among the various other aspects, psychological well-being is a prominent determinant of a fulfilling life in senescence. The present study aims to assess the impact of social isolation and loneliness on the psychological well-being of older adults residing in various old-age homes in India. The study elucidates an insightful evaluation of the prevalence of social isolation and loneliness among residents of old-age homes in the Indian context. It also attempts to understand if social isolation and loneliness affect the different components of psychological well-being differently. Further, the study assays the mediating role of factors like gender, marital status, and education in the association of social isolation and loneliness on the psychological well-being of the older adults.

The findings of the study paints a gloomy picture with almost 84.38% older adults living in old-age homes being socially isolated. Further, almost 86.88% older adults reported to be experiencing higher levels of loneliness. This opinion stands true in the context of other old-age homes in India. Older adults residing in old-age homes are lonelier as compared to their counterparts residing with their families [36, 37]. Our results also align with the findings of Taylor et al., (2018) who opined that almost 70% elderly residing in old-age homes or senior housing communities reported to be experiencing moderate or severe levels of loneliness [38] Results indicate that social isolation predict loneliness significantly stating their co-occurrence to a large extent [39]. Older adults staying in old-age homes may have less social support from management as well as family members and have diminished opportunities to engage themselves in various occasions or programs, therefore leading to heightened levels of social isolation and loneliness [40]. In order to cater to the needs of the older adults and ensure family support, the Ministry of Social Justice and Empowerment, Govt. of India, has enacted the Maintenance and Welfare of Parents and Senior Citizens Act, 2007, bestows upon the older adults with the right to receive adequate maintenance in the form of food, shelter, clothing, and medical facilities. It also directs children and relatives who inherited the property of older adults to take care of them, failing which, they are punishable with imprisonment of three years or monetary fine of INR 5000. Adequate implementation of these provisions can result in decreased levels of social isolation and loneliness among older adults [41].

Furthermore, the study assesses of the impact of objective and subjective dimensions of social interactions on the psychological well-being of older adults residing in old-age homes which demonstrates an increasing trend in the changing socio-cultural context of India. Results reveal that both social isolation and loneliness have a significant impact on the psychological well-being of the older adults. Socially isolated and lonely older adults exhibit poorer psychological well-being as compared to their counterparts who do not experience heightened levels of social isolation and loneliness. Interpersonal relationships have shown significant positive associations with psychological well-being of individuals [42]. Therefore, reduced objective social interactions as well as lessened perceived social ties that foster a greater sense of companionship, can be considered as causal factors for diminished psychological well-being among the elderly. Our results align with the research evidence from other old-age homes in India, which suggest that residents of old-age homes have lowered levels of psychological well-being as compared to elderly staying with families [43]. Further, our findings are concurrent with the findings of Birditt et al. (2021), and Rook (2015), who opine that social isolation and loneliness are predictive of deteriorated psychological well-being among the older adults [44, 45]. Reduced independence to move out of the old-age homes, because of deterred physical health, lack of resources can increase the feelings of social isolation and loneliness, thereby affecting the psychological well-being of the inmates. Moreover, social isolation and loneliness have been reported to have significant impact across all individual components of psychological well-being. However, social isolation has been seen to report no significant impact on the environmental mastery component of psychological well-being unlike the findings of Hausler et al., (2017) who reported a strong association between environmental mastery and social isolation [46]. Individuals residing in old age homes move out of the comfort of their homes, and a lifestyle they have been leading for years, and try to adapt in a new scenario with limited resources which decreases their sense of ability to manage or control various aspects around them. To ensure a fulfilling senescence, the IPSrC scheme which is a sub scheme of National Action Plan for Senior Citizens (NAPSrC) under the Maintenance and Welfare of Parents and Senior Citizens Act, 2007, proposes to set up old-age homes in every district with adequate food, clothing, shelter, security, and recreational facilities for the older adults to live a fulfilling life [41], which might be beneficial in enhancing the psychological well-being of the older adults by catering to their needs. However, the adequate implementation of these provisions is a matter of concern. There is a paucity of formal mechanisms in terms of feedback, appraisal, complaints or grievance redressal, that makes it difficult for ensuring the overall well-being of older adults residing in old-age homes [47].

The study further aimed to assess the mediating impact of various socio-demographic factors in the association of social isolation and loneliness on the psychological well-being of the participants. It was assumed that socio-demographic factors like gender, education, and marital status might mediate the way social isolation and loneliness impact psychological well-being. It was observed that education as an independent factor predicts psychological well-being significantly. The results suggest that education fosters a sense of control among the elderly, and prevents decline in psychosocial functioning. It also helps them become resilient against changes that come with lowered sense of control and hopelessness that are experienced by aging individuals, thereby having a positive impact on the psychological well-being of the aging population [48]. However, gender did not have a significant impact on the psychological well-being of the older adults. The findings are not concurrent with some earlier research evidence, where Matud et al., (2020) opined that men exhibit better psychological well-being particularly in the dimensions of greater self-acceptance, purpose in life, autonomy, and environmental mastery, as compared to women [49]. Older women often experience increased functional impairment, lowered levels of self-reported health, lack of satisfaction with social contacts and perceived social support, and decrease in participation in social activities. All these factors are related to low levels of psychological well-being [50]. However, our results align with earlier research, and the gender differences did not have a significant impact while determining their psychological well-being of the study participants who are residents of old-age homes [51].

Further it was observed that marital status partially mediated the impact of social isolation and loneliness on the psychological well-being of the older adults. Research evidence has highlighted the importance of a spouse for reduced distress and enhanced psychological well-being, particularly in the later years of life [52]. Additionally, Cheng, et al., (2021), have opined that widowhood results in deteriorated subjective well-being, thereby citing the importance of marital status in the declining years of life [53]. Furthermore, research evidence advocates in favour of marital status being an important determinant for reduced levels of loneliness and social isolation [54]. Our results align with the earlier findings. Though social isolation and loneliness seem to impact psychological well-being significantly in a negative way, the impact is reduced in case of married older adults, suggesting the importance of social support in form of marital relationship. Although marital status partially mediated the impact of social isolation and loneliness on psychological well-being of the participants, there was no direct impact of marital status on psychological well-being. In case of the study participants who were married, it was observed that marital status as an independent construct did not act as a protective factor for their psychological well-being. As the older adults are residing in old-age homes and are living apart from their partners despite being married, therefore marital status as an independent factor might not suffice to serve as a protective agent for psychological well-being. The finding is further substantiated by Hank and Wagner (2013), who opine that marital status only when characterized by reciprocity ensures high levels of psychological well-being [55]. Further, the results suggests that irrespective of the gender, level of education, and marital status of the elderly, social isolation and loneliness have a negative association with the psychological well-being of older adults.

## Conclusion

The study comprehensively assesses the impact of social isolation and loneliness on the psychological well-being of the older adults residing in old age homes in India. The findings of the study can be considered to understand the voids resulting from increased social isolation and loneliness among older adults who are residents of old-age homes. The inferences will provide an insight in developing intervention strategies to minimize the feelings of social isolation and loneliness, as well as enhance psychological well-being among them. Increasing social network usage, engaging them in various leisure activities can help in alleviating feelings of social isolation and loneliness. Further, provision of necessary resources to interested and able individuals to engage in various vocations can also help in enhancing a sense of control and autonomy, thereby reducing the feelings of hopelessness, and enhancing psychological well-being among the elderly. Policies aimed to ensure an optimal functioning of the elderly can be framed by incorporating the findings. Based on the findings of the study, the government can incorporate various measures in policies aimed to improve psychological well-being of the elderly. Designing measures where the older adults to empower them with digital technology can aid in reduction of social isolation and loneliness as well as enhance psychological well-being among older adults. Further, providing older adults with opportunities to interact in various social groups to impart their knowledge, like teaching in orphanages, can help in enhancing their overall quality of life. Though the study is first of its kind in the Indian context and holds relevance in the current social scenario given the number of old-age homes increasing at a rapid rate, the study has certain limitations. The study has not considered the physical health status and economic status, which might have an impact on the psychological well-being of the participants. Further, collecting data from various states may help in assessing the impact of varied socio-cultural contexts on psychological well-being within India. Future studies can consider a longitudinal analysis for the above-mentioned factors to gain an insight into the temporal aspects about the impact of social isolation and loneliness on the psychological well-being of the older adults.

## Data Availability

Data sharing is not applicable to this article yet as it is a part of an on-going doctoral research. The dataset used and/or analysed during in the article are available from the corresponding author on reasonable request.

## Declarations

We confirm that all methods, including ethics approval and consent to participate were performed in accordance with the relevant guidelines and regulations or in accordance with the Declaration of Helsinki.

## Abbreviations

PERMA: Positive Emotion, Engagement, Relationships, Meaning, and Accomplishment
LSNS- 6: Lubben Social Network Scale-6

## References

[1] Cunningham C, O’Sullivan R, Caserotti P, Tully MA (2020). Consequences of Physical Inactivity in older adults: a systematic review of reviews and meta-analyses. Scand J Med Sci Sports.

[2] Hedden T, Gabrieli JD (2004). Insights into the ageing mind: a view from cognitive neuroscience. Nat Rev Neurosci.

[3] Wilson RS, Beckett LA, Barnes LL, Schneider JA, Bach J, Evans DA, Bennett DA (2002). Individual differences in rates of change in cognitive abilities of older persons. Psychol Aging.

[4] Theeke LA (2009). Predictors of loneliness in US adults over age sixty-five. Arch Psychiatr Nurs.

[5] Patzelt C, Heim S, Deitermann B, Theile G, Krauth C, Hummers-Pradier E, Walter U (2016). Reaching the Elderly: understanding of health and preventive experiences for a tailored approach–results of a qualitative study. BMC Geriatr.

[6] Holt-Lunstad J, Steptoe A (2022). Social isolation: an underappreciated determinant of physical health. Curr Opin Psychol.

[7] Barnes TL, MacLeod S, Tkatch R, Ahuja M, Albright L, Schaeffer JA, Yeh CS (2022). Cumulative effect of loneliness and social isolation on health outcomes among older adults. Aging Ment Health.

[8] Leigh-Hunt N, Bagguley D, Bash K, Turner V, Turnbull S, Valtorta N, Caan W (2017). An overview of systematic reviews on the public health consequences of social isolation and loneliness. Public Health.

[9] Landeiro F, Barrows P, Musson EN, Gray AM, Leal J (2017). Reducing social isolation and loneliness in older people: a systematic review protocol. BMJ open.

[10] Grenade L, Boldy D (2008). Social isolation and loneliness among older people: issues and future challenges in community and residential settings. Aust Health Rev.

[11] Perlman D, Peplau LA (1981). Toward a social psychology of loneliness. Personal Relationships.

[12] Ong AD, Uchino BN, Wethington E (2016). Loneliness and health in older adults: a mini-review and synthesis. Gerontology.

[13] United Nations, Department of Economic and Social Affairs, Population Division. World Population prospects 2019: Data Booklet (ST/ESA/SER.A/424). New York, United Nations; 2019.

[14] NSO, Elderly in India, National Statistical Office, Ministry of Statistics & Programme Implementation, Government of India, New Delhi. 2021.

[15] Kumar M, Ruikar M, Surya VL. Prevalence and determinants of social isolation among elderly in an urban slum of Raipur city—A community based cross-sectional study. Int J Geriatr Psychiatry. 2022;37(9). 10.1002/gps.5797. 10.1002/gps.5797 35962476

[16] Grover S, Verma M, Singh T, Dahiya N, Nehra R (2019). Loneliness and its correlates amongst elderly attending non-communicable Disease rural clinic attached to a tertiary care centre of North India. Asian J Psychiatry.

[17] Berg-Weger M, Morley JE (2020). Loneliness in old age: an unaddressed health problem. J Nutr Health Aging.

[18] Maher AC, Kielb S, Loyer E, Connelley M, Rademaker A, Mesulam MM, Rogalski E. Psychological well-being in elderly adults with extraordinary episodic memory. PLoS ONE. 2017;12(10). 10.1371/journal.pone.0186413. 10.1371/journal.pone.0186413 PMC5653294 29059208

[19] Seligman ME, Flourish. A visionary new understanding of happiness and well-being. Simon and Schuster; 2012. Feb 7.

[20] Kearns A, Whitley E, Tannahill C, Ellaway A (2015). Loneliness, social relations and health and well-being in deprived communities. Psychol Health Med.

[21] Uchino BN, Cacioppo JT, Kiecolt-Glaser JK (1996). The relationship between social support and physiological processes: a review with emphasis on underlying mechanisms and implications for health. Psychol Bull.

[22] Steptoe A (2019). Happiness and health. Annu Rev Public Health.

[23] Llewellyn DJ, Lang IA, Langa KM, Huppert FA (2008). Cognitive function and psychological well-being: findings from a population-based cohort. Age Ageing.

[24] Tariq J, Zakar R, Ali MV, Zakar MZ, Sajjad A, Fischer F (2023). Determinants of physical, psychological, and social well-being in older adults: a cross-sectional study in senior care facilities of Pakistan (2019/20). BMC Geriatr.

[25] Bekhet AK, Zauszniewski JA (2012). Mental health of elders in retirement communities: is loneliness a key factor?. Arch Psychiatr Nurs.

[26] Sujini SP, Bilquis (2018). A study on Psychological Well Being of Elderly Living in Institutional and non – institutional settings. Int J Pure App Bioscience.

[27] Rajesh MC, OLD AGE HOME, AND ITS IMPACT ON SOCIETY. Int J Creative Res Thoughts. 2022;10(10).

[28] Ministry of Social Justice & Empowerment. Old age homes. 2022 Aug 02. PIB Delhi. Release ID: 1847439.

[29] Barreto M, Victor C, Hammond C, Eccles A, Richins MT, Qualter P (2021). Loneliness around the world: age, gender, and cultural differences in loneliness. Pers Indiv Differ.

[30] Umberson D, Lin Z, Cha H (2022). Gender and social isolation across the life course. J Health Soc Behav.

[31] Amonkar P, Mankar MJ, Thatkar P, Sawardekar P, Goel R, Anjenaya S (2018). A comparative study of health status and quality of life of elderly people living in old age homes and within family setup in Raigad District. Maharashtra Indian Journal of Community Medicine: Official Publication of Indian Association of Preventive & Social Medicine.

[32] Akbar S, Tiwari SC, Tripathi RK, Kumar A, Pandey NM (2014). Reasons for living of elderly to in old age homes: an exploratory study. Int J Indian Psychol.

[33] Lubben JE (1988). Assessing social networks among elderly populations. Family and Community Health.

[34] Russell DW (1996). UCLA Loneliness Scale (Version 3): reliability, validity, and factor structure. J Pers Assess.

[35] Ryff CD, Keyes CL (1995). The structure of psychological well-being revisited. J Personal Soc Psychol.

[36] Jamwal N (2016). Psychosocial consequences among Elderly Living in Institutional and non-institutional settings. Int J Indian Psychol.

[37] KR (2022). Loneliness and self esteem among Elderly Residing In Old Age homes. IOSR J Humanit Social Sci.

[38] Taylor HO, Wang Y, Morrow-Howell N (2018). Loneliness in senior housing communities. J Gerontol Soc Work.

[39] Hwang TJ, Rabheru K, Peisah C, Reichman W, Ikeda M (2020). Loneliness and social isolation during the COVID-19 pandemic. Int Psychogeriatr.

[40] Gonyea JG, Curley A, Melekis K, Levine N, Lee Y (2018). Loneliness and depression among older adults in urban subsidized housing. J Aging Health.

[41] The maintenance and welfare of parents and senior citizens. (amendment) Bill, 2019 [Internet]. 2023 [cited 2023 Sept 7]. Available from: https://prsindia.org/billtrack/the-maintenance-and-welfare-of-parents-and-senior-citizens-amendment-bill-2019.

[42] Hryhorivna OH, Spivak LM (2018). Psychological well-being of elderly people: the social factors. Social Welfare: Interdisciplinary Approach.

[43] Jose JP, Shanuga C (2015). Social Integration and Psychological Well-being of Elderly women in India: a comparative study of elder women at Homes and in elder care facilities. Int J Social Sci.

[44] Birditt KS, Turkelson A, Fingerman KL, Polenick CA, Oya A (2021). Age differences in stress, life changes, and social ties during the COVID-19 pandemic: implications for psychological well-being. Gerontologist.

[45] Rook KS (2015). Social networks in later life: weighing positive and negative effects on health and well-being. Curr Dir Psychol Sci.

[46] Hausler M, Strecker C, Huber A, Brenner M, Höge T, Höfer S (2017). Distinguishing relational aspects of character strengths with subjective and psychological well-being. Front Psychol.

[47] Harbishettar V, Gowda M, Tenagi S, Chandra M (2021). Regulation of long-term care homes for older adults in India. Indian J Psychol Med.

[48] Mitchell UA, Ailshire JA, Brown LL, Levine ME, Crimmins EM (2018). Education and psychosocial functioning among older adults: 4-year change in sense of control and hopelessness. The Journals of Gerontology: Series B.

[49] Matud MP, Bethencourt JM, Ibáñez I, Fortes D (2020). Gender and psychological well-being in older adults. Int Psychogeriatr.

[50] Cummings SM (2002). Predictors of psychological well-being among assisted-living residents. Health Soc Work.

[51] Hafeez A, Rafique R. Spirituality and religiosity as predictors of Psychological Well-being in residents of Old homes. Dialogue (Pakistan). 2013;8(3).

[52] Wright MR, Brown SL (2017). Psychological well-being among older adults: the role of partnership status. J Marriage Family.

[53] Cheng X, Li X, Liu H, Cosco TD, Duan W (2021). Widowhood and the subjective well-being of older people in China: the mediating effects of lifestyle. Appl Res Qual Life.

[54] Štípková M (2021). Marital status, close social network and loneliness of older adults in the Czech Republic. Ageing Soc.

[55] Hank K, Wagner M (2013). Parenthood, marital status, and well-being in later life: evidence from SHARE. Soc Indic Res.

